# Clinical Effectiveness of an Anesthesiologist-Administered Intravenous Sedation Outside of the Main Operating Room for Pediatric Upper Gastrointestinal Endoscopy in Thailand

**DOI:** 10.1155/2010/748564

**Published:** 2010-08-02

**Authors:** Somchai Amornyotin, Prapun Aanpreung

**Affiliations:** ^1^Department of Anesthesiology and Siriraj GI Endoscopy Center, Faculty of Medicine Siriraj Hospital, Mahidol University, Bangkok 10700, Thailand; ^2^Department of Pediatric and Siriraj GI Endoscopy Center, Faculty of Medicine Siriraj Hospital, Mahidol University, Bangkok 10700, Thailand

## Abstract

*Objectives*. To review our sedation practice and to evaluate the clinical effectiveness of an anesthesiologist-administered intravenous sedation outside of the main operating room for pediatric upper gastrointestinal endoscopy (UGIE) in Thailand. *Subjects and Methods*. We undertook a retrospective review of the sedation service records of pediatric patients who underwent UGIE. All endoscopies were performed by a pediatric gastroenterologist. All sedation was administered by staff anesthesiologist or anesthetic personnel. *Results*. A total of 168 patients (94 boys and 74 girls), with age from 4 months to 12 years, underwent 176 UGIE procedures. Of these, 142 UGIE procedures were performed with intravenous sedation (IVS). The mean sedation time was 23.2 ± 10.0 minutes. Propofol was the most common sedative drugs used. Mean dose of propofol, midazolam and fentanyl was 10.0 ± 7.5 mg/kg/hr, 0.2 ± 0.2 mg/kg/hr, and 2.5 ± 1.2 mcg/kg/hr, respectively. Complications relatively occurred frequently. All sedations were successful. However, two patients became more deeply than intended and required unplanned endotracheal intubation. *Conclusion*. The study shows the clinical effectiveness of an anesthesiologist-administered IVS outside of the main operating room for pediatric UGIE in Thailand. All complications are relatively high. We recommend the use of more sensitive equipments such as end tidal CO_2_ and carefully select more appropriate patients.

## 1. Introduction

With the availability of newer and smaller endoscopes, the utilization of endoscopy to diagnose gastrointestinal disorders in children is increasing. Pediatric upper gastrointestinal endoscopy (UGIE) can be completed without sedation, by using intravenous sedation, or with general anesthesia [1–4]. However, the ideal method for sedating children for UGIE remains controversial. 

 Various medication combinations have been used for pediatric sedation, including intravenous ketamine, propofol, midazolam, fentanyl, and pethidine [2]. The standard sedation practice at our institution dependeds on the staff anesthesiologist. The goals of sedation are to ensure patient safety, provide analgesia and amnesia, control behavior during the procedure, enable successful completion of the procedure, and quickly return the patient to pretreatment level of consciousness. 

 In a developing country like Thailand, pediatric UGIE is being performed at increasing rate [5–7]. In addition, in provincial or community hospitals, general anesthesia in the main operating room remains the sedation plan of choice for pediatric UGIE. At Siriraj hospital, a World Gastroenterology Organization (WGO) Endoscopy Training Center, there is a dedicated gastrointestinal endoscopy unit and dedicated anesthesiology service for the unit. Over the years, we have observed a change in the trend of sedation for pediatric UGIE towards intravenous sedation (IVS) technique [5–7]. This study, therefore, is done to review our sedation practice and to evaluate the clinical effectiveness of an anesthesiologist-administered intravenous sedation outside of the main operating room for pediatric upper gastrointestinal endoscopy in Thailand.

## 2. Subjects and Methods

This retrospective study was approved by the Institutional Review Board of the Faculty of Medicine Siriraj Hospital, Mahidol University. All pediatric patients scheduled for UGIE procedures consecutively from March 2006 to October 2009 at the WGO Endoscopy Training Center in Siriraj Hospital were included. Due to hospital policy, all children undergoing GIE were admitted prior to the procedure. All patients who underwent UGIE procedures with IVS were included for analysis. Exclusion criteria were the patients who had hemodynamic instabilities and the patients who needed endotracheal intubation. All sedations for UGIE were clinically titrated to either moderate or deep sedation as defined according to American Academy of Pediatrics and American Academy of Pediatric Dentistry [4]. 

 For all patients who underwent IVS, appropriate monitoring was used. Cardiovascular monitoring included continuous electrocardiogram, heart rate, oxygen saturation measurements and five-minute interval noninvasive blood pressure measurements from blood pressure cuff device. All patients received supplemental oxygenation at 2 L/minute through nasal canula. Ventilation monitoring included continuous respiratory rate measurements and interval observation of patterns of respiration, chest movement, and signs and symptoms of airway obstruction. Level of consciousness was also periodically assessed. End-tidal carbon dioxide (CO_2_) monitoring with capnography or precordial stethoscope was not used during sedation. 

 The following data was obtained: age, gender, weight, ASA physical status, indications, presedation problems, successful completion of the procedure, sedation time, type of intervention, and sedative agents. The presedation problems were defined as the underlying diseases such as cardiovascular disease, hematologic disease and liver disease. The effectiveness of intravenous sedation was defined as successful completion of the procedure at the target sedation level as intended. The secondary outcome variables were complications during and immediately after the procedure. Complications were recorded including: hypotension (defined as a decrease of blood pressure by 20% from baseline and below normal for age), hypertension (defined as an increase of blood pressure by 20% from baseline and above normal for age), bradycardia (defined as a decrease in heart rate by 30% from baseline and below normal for age), and hypoxia (defined as oxygen desaturation with SpO_2 _< 90%). Serious complication is any adverse event not easily treated or managed with medication and/or maintenance of the patient's airway resulting in endotracheal intubation including apnea and/or laryngospasm. 

 Results with variable data were expressed as mean ± SD. Results with categorical data were expressed as percentage (%). Comparison of adverse events by ASA physical status or different medication groups was done by using Student *t*-test. The statistical software package SPSS for Window Version 11 (SPSS Inc., Chicago, IL) was used to analyze the data. A significance level of 5% was used throughout the study.

## 3. Results

During the study period, a total of 168 patients (94 boys and 74 girls), with age ranging from 4 months to 12 years, underwent 176 GIE procedures with IVS. Of these, 26 UGIE procedures were performed with general anesthesia (GA), and 142 UGIE procedures were performed with intravenous sedation (IVS) and reviewed. All sedation was given by a staff anesthesiologist or the anesthetic personnel directly supervised by a staff anesthesiologist physically present in the endoscopy room. Anesthetic personnel included second-year residents in the Anesthesiology residency program and anesthetic nurses who are well-trained in general anesthesia, intravenous sedation, airway management including intubation, and cardiopulmonary resuscitation. There were no premedications prior to the procedure. A single anesthesiologist sedated or supervised the sedation of the patients throughout the study. The equipment used for the procedures included appropriate standard pediatric endoscopes, depending on patient age and size. All endoscopic procedures were performed by a pediatric gastroenterologist. 

 Patient characteristics, duration of sedation, indication of procedure, and the type of interventions are listed in Table 1. Hematologic disease, mild to moderate anemia (40.1%), liver disease, cirrhosis, portal hypertension (37.9%), and electrolyte imbalances, hypo/hyperkalemia and/or hyponatremia (12.4%) were the most common presedation problems. A total of 142 procedures, anesthesiology residents involved in 74 procedures (52.1%), and anesthetic nurses involved in 68 procedures (47.9%).

Table 2 showed the intravenous sedative agents used by age and ASA physical status. Propofol was the most common sedative drugs used in all age and ASA physical status groups. Mean dose of propofol (mg/kg) used in all age groups was significantly different (*P* = .032). However, mean dose of propofol (mg/kg) used in both ASA physical groups was not significantly different (*P* = .365). Additionally, mean dose of fentanyl (mcg/kg), midazolam (mg/kg) and ketamine (mg/kg) in all age and ASA physical status groups was not significantly different. However, the number of fentanyl used in the 0–2.99 years-old group (60.0%) was relatively lower than in the other groups (86.5% and 87.5%). Nevertheless, the number of ketamine used in the 0–2.99 years-old group (75.0%) was significantly higher than in the other groups (39.2% and 16.7%). According to ASA physical status, there were no significant differences in the number of propofol, fentanyl, midazolam and ketamine used. 

 There were no failures of sedation. However, two patients became more deeply than intended and required unplanned endotracheal intubation. These two patients were 5-month and 7-month old. They were then intubated. After the patient's status had improved, the procedure was completed with GA. 

Table 3 showed the sedation related-complications comparing ASA physical status groups. Overall, 36 patients (25.4%) experienced sedation related complications. Respiratory complications with hypoxia occurred in seven patients (4.9%), and upper airway obstruction occurred in six patients (4.2%). Cardiovascular complications arose in 23 patients (16.2%) and mainly consisted of hypotension (14 patients) and bradycardia (9 patients). If only serious complications are included, the complication rate is none. All complications were easily treated and managed with medication and/or maintenance of the patient's airway by the staff anesthesiologist or anesthetic personnel under direct supervision of a staff anesthesiologist who was physically present in the room. There was no difference in the incidence of complications when sedated by trainees, anesthetic nurses, or anesthesiologist. 

 The overall complications in children who had ASA physical status I-II as compared to ASA physical status III-IV were not significantly different (*P* = .202). Similarly the respiratory and cardiovascular complications between these two groups were not statistically different. In addition, one patient in ASA physical status I-II and one patient in ASA physical status III-IV developed hypoxia and hypotension. Two patients in ASA physical status I-II and one patient in ASA physical status III-IV developed upper airway obstruction and bradycardia. The emergence reactions or hallucinations, increased salivation or laryngospasm were not seen in patients receiving ketamine as part of IVS. 

## 4. Discussion

This retrospective study demonstrates the clinical effectiveness of an anesthesiologist-administered intravenous sedation outside of the main operating room for pediatric upper gastrointestinal endoscopy in a developing country. The complication rate of our study is relatively high. However, the serious complications were none. IVS for pediatric UGIE procedure in children 12 years of age and younger is challenge and requires an experienced anesthesiologist as well as appropriate monitoring. Anesthetic personnel should remind themselves to use more sensitive equipments to detect potential complication such as end tidal CO_2_ and carefully select more appropriate patients. 

 UGIE procedure in children is an important and effective tool for the diagnosis and treatment of upper digestive tract diseases. The indications for upper endoscopy in the pediatric age group are similar to those for adult endoscopy [8]. These procedures are generally performed either with IVS in the endoscopy room, or under GA in the operating room [9]. The decision to use GA is usually based on the patients' parameters such as age, diagnosis, respiratory compromise and severity of disease. In some centers, GA is used on all infants, children and adolescents [3, 10, 11]. However, in other centers, IVS is used for the procedures. With IVS, several medication combinations have been used successfully [9, 12–15]. 

 In a developing country where pediatric UGIE performed at increasing rates, the majority of cases are performed under general anesthesia in the operating room (OR). At Siriraj Hospital, there is a dedicated endoscopy unit with dedicated anesthesia service. Over the last two years, 2006 to 2008, we performed most pediatric UGIE with IVS [5–7]. We followed the guidelines provided by the American Academy of Pediatrics and American Academy of Pediatric Dentistry and ASA standards for sedation providers [4, 16]. Our previous reviews of IVS practice in pediatric population showed that it can be done safely with various sedative combinations with proper monitoring and anesthesiology service supervision.

 Majority of children received propofol in combination with other sedatives. Propofol has gained wide acceptance among adult gastroenterologist. Its use in pediatric population has been shown to be safe, effective and reliable [10–15]. In Thailand, sedation with propofol is administered by anesthesiologist. The drug combination provides synergistic action while lowering the doses of each agent. Our practice reflects this where many different combination regimens were used [5–7]. Propofol is the most common agent used in combination with midazolam and fentanyl in this study. Additionally, we did not observe hemodynamic instability, emergence reactions, hallucinations, increased salivation or laryngospasm with the use of ketamine combining regimen. This observation was similar in the previous studies [17–19].

 Cardiopulmonary complications account for more than half of the major complications during endoscopy, and are often related to hypoxia, especially in children less than 1 year old [20, 21]. In our study, the overall adverse event was relatively high (25.4%). Cardiovascular complications accounted for the majority (16.2%) followed by respiratory complications (9.2%). However, all complications were transient and easily treated with no adverse sequelae. Many previous studies involving the use of propofol and other combination sedative drugs have reported slightly higher adverse events [22–24]. In our study, there was significant difference in the mean dose of propofol between the three aged groups.

 In a study by Barbi and colleagues, major desaturation was noted in 0.7% of all the children, and transient desaturation that resolved sponstaneously occurred in 12% of all the procedures [22]. Additionally, the study by Yildizdaş et al. demonstrated that the use of propofol and midazolam/fentanyl in 126 children had 16.6% incidence of respiratory depression as shown by high end-tidal carbon dioxide (>50 mmHg) [23]. The high incidence of respiratory depression reflected the better detection of respiratory depression by the use of end-tidal carbon dioxide. In our study, complication rate is comparable to studies that did not use end-tidal carbon dioxide monitoring [22, 24]. ASA physical status III-IV has been shown to be a predictor of increased risk for sedation-related complications [24]. There is also a concern for increased respiratory complication in patients undergoing UGIE procedures. Endoscope can potentially compress and obstruct airway. 

 Several publications described the use of propofol for sedation by physicians or providers other than anesthesiologists [24–27]. Consequently, there was a difference in outcomes once nonanesthesiologists use propofol. When a dedicated pediatric sedation team involving an anesthesiologist was utilized, the reported successful sedation rates were 100%, and adverse events ranged from 1.7 to 5% [28]. There was no failure of sedation in this study. However, two patients became more deeply than intended and required unplanned endotracheal intubation. Finally, all procedures were completed as intended. A high success rate in our study is due to the procedure is performed by an experienced endoscopist and is sedated by an experienced anesthesiologist. Consequently, our center had a dedicated anesthesia service involved with sedation and the use of basic noninvasive monitoring, which includes noninvasive blood pressure monitoring, pulse oximetry, and electrocardiogram. Additionally, the safe and successful sedation is also dependent on proper preparation, evaluation, monitoring, and appropriate skills to rescue the patient, and proper recovery [27]. 

 Our study has several limitations. This is a retrospective paper of a cohort of patients undergoing pediatric UGIE with IVS. We accept that there are limitations with chart review in regards to proper and complete documentation. We also realized that with this review, the study is reflected in the variety of regimen and sedative drugs used for IVS. In addition our cohort varied widely in age range. Therefore, the drug requirement, drug doses, and side effects varied as well. According to the design of study, we defined an alteration of blood pressure by 20% from baseline, and a decrease in heart rate by 30% from baseline as the complication. The complication rate in this paper was also relatively high. Moreover, we did not use the end tidal CO_2_ monitoring. Overall, even with these limitations, we believe that the study findings are applicable to the sedation practice and to remind the physicians for sedation the pediatric patients for UGIE procedures. 

 In summary, this study shows the clinical effectiveness of an anesthesiologist-administered intravenous sedation outside of the main operating room for pediatric upper gastrointestinal endoscopy in a developing country. Although, the complication rate of our study is relatively high. All complications were transient and easily treated with no adverse sequelae. We also recommend the use of more sensitive equipments to detect potential complication such as end tidal CO_2_ and carefully select more appropriate patients for pediatric UGIE. 

## Figures and Tables

**Table 1:** Patient characteristics, duration of sedation, and indication of procedure.

Variable	Overall (*n* = 142)
Age (yr) (mean, SD; range)	7.2 (3.7); 0.04–12.0
Gender (Male/Female; %)	80/62 (56.3/43.7)
Weight (kg) (mean, SD; range)	23.5 (11.3); 2.7–55
ASA physical status (I/II/III/IV; %)	43/47/50/2 (30.3/33.1/35.2/1.4)
Duration of sedation (min) (mean, SD; range)	23.2 (10.0); 5.0–60.0
Indication of procedure	
Variceal screening	40 (28.2)
Abdominal pain	27 (19.0)
History of upper gastrointestinal hemorrhage	16 (11.3)
Chronic vomiting	10 (7.0)
Anemia	9 (6.3)
Others	40 (28.2)
Type of intervention	
Diagnostic procedure	99 (69.7)
Therapeutic procedure	43 (30.3)
Variceal banding	24 (16.9)
Sclerosing injection	16 (11.3)
Esophageal dilatation	2 (1.4)
Remove foreign body	1 (0.7)

**Table 2:** Intravenous sedative agents used by age and ASA physical status.

	0–2.99 yr	3–9.99 yr	>9.99 yr	*P*-value	ASA I-II	ASA III-IV	*P*-value
	(20)	(74)	(48)		(90)	(52)	
Propofol				.032^(a)^			.365
*n* (%)	19 (95.0)	72 (97.3)	46 (95.8)		88 (97.8)	49 (94.2)	
mg/kg (mean, SD)	2.28 (2.29)	3.50 (2.99)	4.43 (3.36)		3.65 (3.44)	3.63 (2.37)	
Fentanyl				.896			.276
*n* (%)	12 (60.0)	64 (86.5)	42 (87.5)		76 (84.4)	42 (80.8)	
mcg/kg (SD, range)	0.96 (0.16)	0.95 (0.20)	0.97 (0.27)		0.96 (0.21)	0.96 (0.25)	
Midazolam				.657			.578
*n* (%)	17 (85.0)	57 (77.0)	38 (79.2)		68 (75.6)	44 (84.6)	
mg/kg (SD, range)	0.05 (0.05)	0.06 (0.07)	0.06 (0.04)		0.06 (0.06)	0.06 (0.04)	
Ketamine				.082			.564
*n* (%)	15 (75.0)	29 (39.2)	8 (16.7)		29 (32.2)	23 (44.2)	
mg/kg (SD, range)	2.68 (4.44)	1.05 (0.24)	0.85 (0.33)		1.10 (0.35)	1.98 (3.66)	

^(a)^considered statistically significant.

**Table 3:** Complications comparing ASA physical status groups.

Complications	ASA I-II (90)	ASA III-IV (52)	*P*-value
(36)	*n* (%)	*n* (%)	
Overall	26 (28.9)	10 (19.2)	.202
Respiratory	10 (11.1)	3 (5.8)	.288
Hypoxia	5 (5.6)	2 (3.8)	.650
(SpO_2_ < 90%)			
Upper airway	5 (5.6)	1 (1.9)	.300
Obstruction			
Cardiovascular	16 (17.8)	7 (13.5)	.501
Hypotension	9 (10.0)	5 (9.6)	.941
Bradycardia	7 (7.8)	2 (3.8)	.354
